# Examining the effectiveness of general practitioner and nurse promotion of electronic cigarettes versus standard care for smoking reduction and abstinence in hardcore smokers with smoking-related chronic disease: protocol for a randomised controlled trial

**DOI:** 10.1186/s13063-019-3850-1

**Published:** 2019-11-28

**Authors:** Rachna Begh, Tim Coleman, Lucy Yardley, Rebecca Barnes, Felix Naughton, Hazel Gilbert, Anne Ferrey, Claire Madigan, Nicola Williams, Louisa Hamilton, Yolanda Warren, Jenna Grabey, Miranda Clark, Anne Dickinson, Paul Aveyard

**Affiliations:** 1 Nuffield Department of Primary Care Health Sciences, University of Oxford, Oxford, OX2 6GG UK; 2 Division of Primary Care, University of Nottingham, Queen’s Medical Centre, Nottingham, NG7 2UH UK; 3 Academic Unit of Psychology, Faculty of Social and Human Sciences, University of Southampton, Southampton, SO17 1BJ UK; 4 School of Experimental Psychology, University of Bristol, Bristol, BS8 1TU UK; 5 Population Health Sciences, Bristol Medical School, University of Bristol, Canynge Hall, 39 Whatley Road, Bristol, BS8 2PS UK; 6 School of Health Sciences, University of East Anglia, Norwich, NR4 7UL UK; 7 Research Department of Primary Care and Population Health, University College London, Royal Free and University College Medical School, London, NW3 2PF UK

**Keywords:** Smoking, Tobacco, Electronic cigarettes, Cessation, Quitting, Addiction, Treatment, Primary care

## Abstract

**Background:**

Despite the clear harm associated with smoking tobacco, many people with smoking-related chronic diseases or serious mental illnesses (SMI) are unwilling or unable to stop smoking. In many cases, these smokers have tried and exhausted all methods to stop smoking and yet clinicians are repeatedly mandated to offer them during routine consultations. Providing nicotine through electronic cigarettes (e-cigarettes) may reduce the adverse health consequences associated with tobacco smoking, but these are not currently offered. The aim of this study is to examine the feasibility, acceptability and effectiveness of general practitioners (GPs) and nurses delivering a brief advice intervention on e-cigarettes and offering an e-cigarette starter pack and patient support resources compared with standard care in smokers with smoking-related chronic diseases or SMI who are unwilling to stop smoking.

**Methods/design:**

This is an individually randomised, blinded, two-arm trial. Smokers with a smoking-related chronic condition or SMI with no intention of stopping smoking will be recruited through primary care registers. Eligible participants will be randomised to one of two groups if they decline standard care for stopping smoking: a control group who will receive no additional support beyond standard care; or an intervention group who will receive GP or nurse-led brief advice about e-cigarettes, an e-cigarette starter pack with accompanying practical support booklet, and telephone support from experienced vapers and online video tutorials. The primary outcome measures will be smoking reduction, measured through changes in cigarettes per day and 7-day point-prevalence abstinence at 2 months. Secondary outcomes include smoking reduction, 7-day point-prevalence abstinence and prolonged abstinence at 8 months. Other outcomes include patient recruitment and follow-up, patient uptake and use of e-cigarettes, nicotine intake, contamination of randomisation and practitioner adherence to the delivery of the intervention. Qualitative interviews will be conducted in a subsample of practitioners, patients and the vape team to garner their reactions to the programme.

**Discussion:**

This is the first randomised controlled trial to investigate whether e-cigarette provision alongside a brief intervention delivered by practitioners leads to reduced smoking and abstinence among smokers with smoking-related chronic diseases or SMI.

**Trial registration:**

ISRCTN registry, ISRCTN59404712. Registered 28/11/17.

## Background

Although smoking has more than halved since the 1970s, 14.7% of adults in the UK still smoke [1], costing the National Health Service (NHS) £2.5 billion a year from treating smoking-related chronic diseases [2]. Reducing smoking remains a key public health priority, particularly among those with smoking-related chronic diseases and serious mental illnesses (SMI), as their health can improve if they stop smoking or decline if they continue. In people with chronic obstructive pulmonary disease (COPD) continued smoking increases the risk of hospitalisation and chest infections and stopping smoking is the only intervention known to decrease the rate of decline in lung function [3, 4]. In hypertension, diabetes and chronic kidney disease, continued smoking leads to more rapid progression of renal disease [5–7]. In asthma, smoking cessation improves symptom control and can decrease the need for medication [8, 9]. In ischaemic heart disease, smoking cessation reduces recurrence by a third, more than any other therapeutic intervention [10]. Stopping smoking is associated with reduced anxiety and stress among people with mental health conditions [11]. Overall, quitting reduces mortality, even in people with smoking-related morbidity [12, 13]. Although almost everyone with a serious smoking-related disease tries to stop smoking on diagnosis [14], relapse is common, with evidence that most smokers continue smoking [15].

People with smoking-related conditions are often called ‘hardcore smokers’ because they have often tried and failed to stop smoking, despite trying most available means [16]. Similarly, smokers with SMI are often defined as hardcore smokers as they tend to smoke more heavily and find it more difficult to quit than those without SMI [17]. Primary care practitioners are well-placed to support these groups in stopping smoking and this is encouraged in general practices in the UK through a ‘pay for performance’ contract called the Quality and Outcomes Framework (QOF) [18]. The QOF rewards GPs for providing quality care for people with smoking-related illnesses and SMI. Since inception, GPs have had to offer advice on smoking, and since 2012, they offer referral for support and pharmacotherapy annually to people with smoking-related conditions and with serious mental health disorders. While the QOF has improved rates of recording advice and referrals [19], some smokers are still less likely to receive help in quitting [20].

There are several reasons why people continue to smoke despite sincere desire not to do so. Tobacco addiction is driven by several factors including social, psychological and environmental influences, but it is the physiological effect of nicotine that underpins smoking behaviour [21]. Nicotine is positively reinforcing through the release of the chemical dopamine in the brain, which produces feelings of pleasure. The rewarding effects of nicotine start to dissipate within hours of the last cigarette smoked, after which smokers experience discomfort in the form of withdrawal symptoms and urges to smoke [22]. Smoking alleviates nicotine withdrawal and so a cycle of continued smoking ensues to ameliorate these symptoms and maintain nicotine levels. With repeated cigarette use, smokers come to associate particular moods, environmental cues or the sensory aspects of smoking with the rewarding effects of nicotine until these smoking-related cues are sufficient to instigate urges to smoke and maintain smoking behaviour in their own right [23].

While people smoke for their need for nicotine, it is the other constituents in tobacco smoke that cause almost all harm [24]. The adverse health consequences of continuing to smoke can largely be avoided if smokers obtain nicotine through less harmful sources than smoking cigarettes. While licensed nicotine replacement therapy (NRT) offers this, electronic cigarettes, known as e-cigarettes, have become the most popular non-tobacco system for obtaining nicotine. Around 32% of smokers are using e-cigarettes to support quit attempts, which is substantially more than those who are using prescription medication (4%), purchasing NRT over the counter (14%) or using NHS Stop Smoking Services (1%) [25, 26]. Of those who smoke and use nicotine from other sources, around 19% use e-cigarettes compared with 7% using NRT [26]. The National Centre for Smoking Cessation Training (NCSCT) recommends that stop smoking practitioners “be open to electronic cigarette use in people keen to try them; especially in those that have tried, but not succeeded, in stopping smoking with the use of licensed stop smoking medicines” [27]. The evidence suggests that e-cigarettes appeal particularly to people who do not want to stop smoking [28]; however, primary care services are unable to prescribe them, as there is no medically licensed e-cigarette currently available in the UK.

While research on the safety and efficacy of e-cigarettes is ongoing, sufficient evidence indicates that they are substantially less harmful than combustible tobacco [29–32], making them particularly suitable for harm reduction [28, 33]. Studies of e-cigarette use in smokers with no intention of quitting have shown promising findings, suggesting they promote reduction and cessation [34–36]. E-cigarettes could appeal because they appear to be about switching not quitting, and thereafter allow people to cut down and eventually stop smoking when they find that the e-cigarette meets their needs. There is strong trial evidence that NRT helps smokers with no immediate intention to quit to reduce and quit in this way [37, 38]. Recent estimates in 2018 suggest that e-cigarettes could be contributing to an additional 22,000–57,000 quitters in England who would not have stopped otherwise [39].

A Cochrane review on e-cigarettes for cessation showed that approximately 9% of smokers who used e-cigarettes were able to stop smoking at up to one year compared with 4% of smokers who used nicotine-free e-cigarettes [39]. In those who did not quit, 36% of e-cigarette users halved the number of conventional cigarettes they smoked compared with 27% of users on placebo. Furthermore, none of the studies found that smokers who used e-cigarettes for two years or less had an increased health risk compared with smokers who did not use e-cigarettes. Since the Cochrane review, a large randomised controlled trial comparing e-cigarettes with NRT alongside behavioural support found an almost two-fold increase in quitters at one year in the e-cigarette group [40]. Another randomised controlled trial found that among smokers who did not want to quit, 34% of those given an e-cigarette quit smoking compared with 0% of those not given an e-cigarette. The group given e-cigarettes also reduced their cigarette consumption substantially more than those who were not given e-cigarettes [41]. While some population studies have detected similar effects on cessation [42, 43] and not all observational studies have done so [44], it is clear that more clinical studies are needed, particularly in smokers who have no intention of quitting.

Several UK health authorities including the National Institute for Health and Care Excellence (NICE), Public Health England and the Royal College of Physicians have stated that primary care physicians should offer advice to smokers about using e-cigarettes as a means to stopping smoking [24, 45, 46]. We know that many physicians are being asked about e-cigarettes by their patients and some are recommending their use [47], but little is known about the type of information that is being communicated and the impact this has on patients’ use. Health professionals appear to be uninformed [48]; smoking cessation practitioners have expressed confusion about e-cigarettes and a desire to provide better information to service users, although brief training can improve knowledge and confidence [49]. If smoking cessation specialists are confused, it is likely that primary care doctors and nurses will be even less aware, drawing their knowledge from stories in the media, many of which feature alarmist and misleading information on the risks of e-cigarettes [45, 50]. Training practitioners on e-cigarettes and their use as a harm reduction approach is therefore important and is addressed in this study.

GP brief interventions on smoking motivate more people to take action when GPs offer help to change rather than advice to do so on medical grounds [51]. Furthermore, there is evidence that providing immediate connection to help rather than leaving the patient to seek it leads to a ten-fold increase in the rate of use of help [52]. GP brief advice is a potent prompt to change patients’ behaviour, which has been found in a trial of a 30-sec GP brief intervention for weight loss [53]. The study team screened for obesity and asked anyone who was willing to join the trial and 83% agreed. In the active intervention, the GP offered help to lose weight and an immediate booking for support. Overall, 77% of overweight patients agreed to a referral to a weight management service and 44% attended. A similar approach will be used here, where GPs and nurses will give brief advice and offer an e-cigarette to hardcore smokers (i.e. those unwilling to quit), with smoking-related chronic diseases or SMI. The theory underpinning this approach comes from Social Cognitive Theory to build on patient and practitioner motivation, skills, positive outcome expectations and self-efficacy [54].

The aim of this study is to examine the feasibility, acceptability and effectiveness of an intervention to encourage hardcore smokers with smoking-related chronic diseases or SMI who are unwilling to quit to switch to e-cigarettes and investigate how this might affect their smoking behaviour compared with standard care. The intervention comprises a brief behavioural intervention by a GP or practice nurse to promote the use of e-cigarettes, providing an e-cigarette starter pack, a practical support booklet, online tutorials and ongoing technical support to use the e-cigarette from experienced vapers.

### Trial objectives

#### Primary objectives

The primary objective is to examine the effectiveness of a brief GP/nurse behavioural intervention to encourage switching to e-cigarettes, provision of e-cigarettes and ongoing technical support from experienced e-cigarette users in producing short-term reductions in cigarette intake and smoking abstinence.

#### Secondary objectives

The secondary objectives are: to examine recruitment and follow-up of patients; to examine smokers’ uptake and use of offered e-cigarettes; to assess contamination of randomisation; to examine nicotine intake; to assess the adherence of primary care teams in delivering brief interventions; to examine practitioners’ reactions towards offering e-cigarettes and experiences of delivering the intervention; to examine patients’ reactions to the programme; to examine the vape team’s reactions towards supporting patients in their use of e-cigarettes; and to examine the effectiveness of a GP/nurse-led brief intervention for smoking on long-term reductions in cigarette intake and smoking abstinence. For more information on the objectives, outcomes and time-points of measures, see Additional file 1.

## Methods/design

### Study design

This is a two-centre, individually randomised, two-arm, parallel group study. The study will take place in general practices in England. Smokers with a smoking-related chronic disease or SMI and who have no intention of stopping immediately or seeking cessation support will be randomised to one of two groups, if they decline referral to NHS stop smoking services (SSS) and smoking cessation medication: an intervention group offered encouragement by their practitioner to use an e-cigarette, or a standard care control group who will receive nothing beyond the usual care already provided prior to randomisation. Participants in the intervention group will be offered an e-cigarette starter pack and accompanying practical support booklet; this will contain details for a telephone call back service run by experienced vapers for technical support and a website with an online video tutorial. Patients will attend four visits at their GP practice: a baseline visit, a therapeutic visit with their GP or nurse and two follow-up visits 2 months and 8 months post-consultation.

### Participant entry

**Fig. 1:**
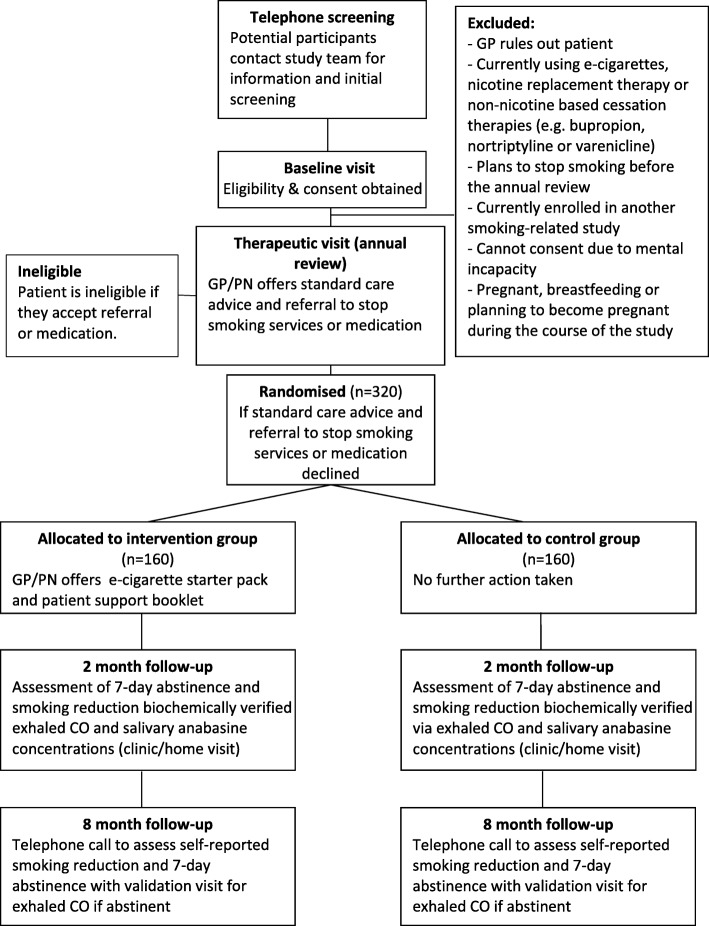
Planned participant flow through the trial

A total of 320 participants will be recruited into the trial: 160 participants in each of the two treatment arms. See Fig. 1 for the planned flow of participants through the trial and Additional file 2 for the SPIRIT checklist.

### Recruitment

Recruitment centres at the Universities of Oxford and Nottingham (United Kingdom) will recruit through GP practices. General practice teams review patients with chronic diseases at least annually, which includes enquiry about smoking status and, for smokers, offer of help to quit smoking. Around 20–30 GP practices will search their patient databases for all current smokers with one or more chronic conditions who are due to have an annual review in the new QOF year, beginning in April 2018. GPs will screen the search list to ensure that all those identified are medically appropriate to participate in the trial. Practice staff will write to or send a text message to these patients inviting them to take part. Patients who are interested in taking part will contact the study team by telephone, text message, email or return reply slip.

The study team will carry out preliminary screening and if the patient appears to be eligible, s/he will be asked to attend a baseline assessment at their GP practice to confirm eligibility (see Additional file 3 for enrolment process). A participant information sheet (PIS; see Additional file 4) and baseline appointment confirmation letter will be sent to potential participants.

### Inclusion criteria

- Participant is willing and able to give informed consent for participation in the study
- Aged 18 years or above
- Current smoker with a value of at least 10 parts per million (ppm) for exhaled carbon monoxide (CO) and smokes a minimum of eight cigarettes/8 g of tobacco per day (including pipe, cigars or tobacco roll-ups)
- Diagnosed with one or more of the following chronic conditions: ischaemic heart disease, peripheral vascular disease, hypertension, diabetes mellitus (type 1 and type 2), stroke, asthma, COPD, chronic kidney disease, depression, schizophrenia, bipolar disorder or other psychoses

### Exclusion criteria

- GP believes that switching to e-cigarettes would not benefit the patient given their current medical condition
- Currently using e-cigarettes, nicotine replacement therapy or other cessation therapies (e.g. bupropion, nortriptyline or varenicline)
- Plans to stop smoking before or at the annual review
- Currently enrolled in another smoking-related study or other study where the aims of the studies are incompatible
- Cannot consent due to mental incapacity
- Pregnant, breastfeeding or planning to become pregnant during the course of the study

### Withdrawal criteria

Each participant has the right to withdraw from the trial at any time. The PIS will provide details on how to withdraw and who to contact should the participant no longer wish to participate in the trial. If the participant wishes to withdraw we will use their data up to the point that they withdrew unless they request that we do not do so. Such patients will not be replaced. There are no withdrawal criteria other than the participant or practitioner’s request to withdraw, which will be dealt with on a case by case basis.

Failure to attend follow-up visits will not count as withdrawal from the trial; patients will be classed as smokers and as not having reduced consumption.

### Randomisation and blinding

Participants will be randomised to intervention or control with a 1:1 allocation ratio. A randomisation list will be generated by the trial statistician using the current version of Stata and validated by a second statistician within the Primary Care Clinical Trials Unit (PC-CTU). The randomisation will be stratified by practice and will use varying block sizes to ensure allocation concealment. The randomisation list will then be passed to someone independent of the trial who will create the randomisation envelopes.

Practitioners will randomise patients during the annual review appointment. On the day of the annual review session, a researcher will give each practitioner a set of opaque A5 sealed envelopes, which will contain a colour-coded randomisation card. The envelopes will be numbered in sequence. The GP or practice nurse will only open the randomisation envelope after they have delivered usual care advice on smoking and are satisfied that the patient meets eligibility for the trial, i.e. the patient confirms that s/he is currently a smoker and declines usual care treatment for stopping smoking. The patient is classed as enrolled in the trial only after the randomisation envelope has been opened. The randomisation card inside the sealed envelope will reveal the patient’s treatment allocation to the GP or nurse, but not the patient. The randomisation envelope will be returned to the researcher by the patient after their consultation.

If the patient is no longer a smoker or accepts usual care treatment, practitioners will fill in a patient ineligibility card containing a tick box option to select if they did not randomise and enrol the patient into the trial. The tick box will indicate the reason for non-enrolment, i.e. patient has stopped smoking, patient accepted usual care or any other reason where it is deemed clinically inappropriate. The ineligibility card will be returned to the researcher after the session and the patient will be thanked for their time given to the study and debriefed.

Due to the nature of the trial, GPs and practice nurses will be aware of the patient’s treatment allocation to ensure that the correct intervention is given. Therefore, practitioners who are delivering the intervention cannot be blinded to treatment. While patients will know whether they have been offered support to cut down by using an e-cigarette or not by their GP or nurse, the patient will not be informed that this study is investigating this specifically and therefore will in some respects remain blind to allocation. All patient-facing materials will therefore refer to the study’s short title, ‘Management of Smoking in Primary Care’ (MaSC), so that the nature of the trial remains concealed.

### Study visits

**Fig. 2:**
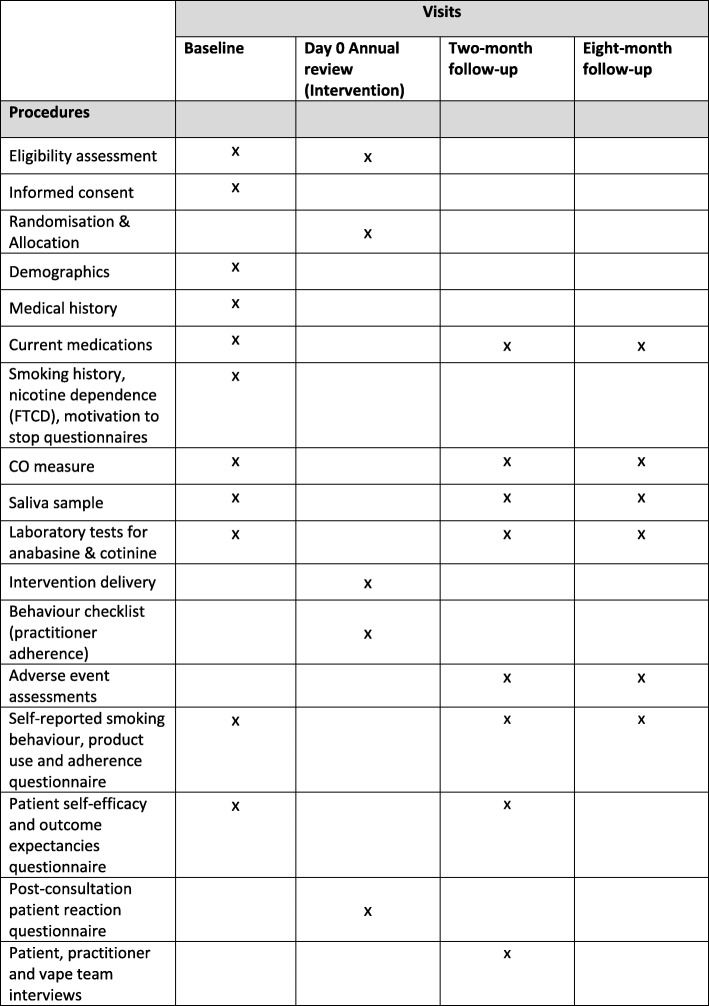
Schedule of study visits, procedures and assessments

Figure 2 provides a summary of participant activities and assessments by study visits.

#### Baseline visit

The baseline visit will take place in the patient’s GP practice. The researcher will assess the patient’s eligibility and obtain written informed consent (Additional file 5). The researcher will check for eligibility based on the patient’s medical history, medication use, current e-cigarette use and smoking status, which will be biochemically verified through exhaled carbon monoxide (CO) readings. Medical history and medication use may be checked against the participant’s primary care records to ensure accuracy and completeness. The researcher will also collect basic demographic data, information on smoking history and a saliva sample. Table 1 outlines the data that will be collected at baseline.

**Table 1:** Data collection at baseline visit

Demographics	Basic demographic information
Medical history	Medical problems and concomitant medication
Smoking history	Information on smoking and quitting history (age when started smoking, number of cigarettes smoked per day, when the last cigarette was smoked, type of cigarettes smoked per day (roll-ups or factory-made), usual pack/pouch size, nicotine dependence, motivation to stop smoking, self-efficacy and outcome expectancies, history of any pharmacotherapy or e-cigarette use and exhaled CO measurement)
Biological samples	Saliva samples to measure anabasine (for smoke intake) and cotinine (for nicotine intake)

#### Therapeutic visit

Participants will be invited to attend an annual review appointment for their chronic condition in the new QOF year, from April 2018. The intervention will be delivered by the GP or practice nurse during this appointment.

A researcher will be present in the GP practice on the day of the participant’s annual review appointment to remind practitioners of the study procedures and to collect data after the consultation. Neither clinicians nor patients are reliable reporters of their communication behaviour [55] and recording does not seem to change consultation content or behaviour [56]. Therefore, audio recordings will be used to monitor adherence to the intervention (including quality of delivery as well as immediate patient responsiveness) and the degree of local tailoring being adopted across practitioners and practices. The recordings will be checked by the study team using a study-specific behaviour checklist.

During the consultation, the practitioner will enquire about the participant’s smoking status. If the participant reports that they no longer smoke, the practitioner will complete a patient ineligibility card and cease to provide any further smoking cessation advice. If the participant confirms that they are a smoker, the practitioner will deliver usual care as outlined below. The GP/practice nurse will open a randomisation envelope if the participant is eligible for the trial, i.e. the participant declines usual care treatment. Participants randomised to the control group will be given no further advice beyond what they have been given (i.e. offered usual care), while the intervention group will be offered an e-cigarette starter pack and support materials. At the end of the consultation, all participants will be asked to return the randomisation card and envelope to a researcher who will be waiting in another room. The researcher will then ask the participant to complete a post-consultation questionnaire to assess the quality of advice given during their consultation and contamination, via recall of key points of the consultation. The consultation audio-recording will be reviewed if the post-consultation questionnaire reveals that contamination has occurred, i.e. participants in the control group recall that e-cigarettes were mentioned by the GP/nurse. If the recording confirms contamination, the researcher will deliver brief refresher training to the practitioners concerned.

#### Two-month follow-up

Study staff will contact participants and schedule a follow-up appointment at the GP practice or carry out a home visit 2 months after randomisation. At this visit, the researcher will record information on the types of medication currently used, and any adverse events. S/he will collect information on smoking behaviour including self-reported smoking status, the number of cigarettes smoked per day, current pack/pouch size, use of e-cigarettes and pharmacotherapy (NRT, varenicline or bupropion) and nicotine dependence. A saliva sample and exhaled CO measurement will be taken by the researcher. S/he will also record whether the participant accessed any type of support (e.g. NHS SSS) for stopping or reducing smoking since randomisation. If the participant reports that they have stopped smoking and they are taking a particular medication that requires a dose adjustment due to this, the participant’s GP will be informed. For intervention group participants, we will assess the frequency of telephone support and use of the online video tutorial. If participants are unable to attend a practice visit or a home visit, information on smoking behaviour will be collected by telephone, text message or email. Salivettes will be posted out to participants with instructions on how to give a saliva sample and a pre-paid envelope will be provided for returning the sample back to the study team.

#### Eight-month follow-up

The study team will contact all participants 6 months after their 2-month follow-up by telephone, text message or email to establish smoking status, number of cigarettes smoked per day, current pack/pouch size, use of pharmacotherapy, use of e-cigarettes and any serious adverse events (SAEs). Participants who declare abstinence will be invited to return to the practice or receive a home visit by a member of the study team for exhaled CO measurement to confirm this. Every effort will be made to contact participants exactly 6 months after the 2-month follow-up, before data are classed as lost to follow-up. If the participant is unable to provide a CO measurement to validate abstinence, a salivette will be posted to the participant with instructions on how to return the sample back to the study team. Again, if the participant reports that they have stopped smoking and they are taking a particular medication that requires a dose adjustment, the participant’s GP will be informed.

### Treatments

#### Treatment arms

##### Control

Practitioners will deliver routine smoking cessation advice and treatment to all patients. Although usual care varies by practice, this typically involves standard brief advice about stopping smoking and assistance to do so either by referral to the NHS SSS or offer of pharmacotherapy. Some practices offer in-house SSS delivered by nurses or healthcare assistants trained to provide stop smoking support. In some practices, practitioners prescribe cessation medication. Pharmacotherapies provided as part of this support by the practitioner or through the SSS referral include either nicotine replacement therapy (used as single form or combination NRT), varenicline or bupropion. If the patient agrees to a referral to the SSS or accepts a prescription for pharmacotherapy, the GP/practice nurse will fill in a patient ineligibility card. Practitioners will only open the randomisation envelope if the patient declines usual care treatment. If the randomisation card reveals that the patient is allocated to the control arm, no further action will be necessary as the GP/practice nurse has delivered all components of usual care.

##### Intervention

Participants in the intervention group will have already received usual care by the GP or practice nurse as described above for the control group. If randomised to the intervention group, the GP or practice nurse will deliver brief advice about e-cigarettes. All practitioners will receive online training in how to deliver the advice and motivate patients to take up the offer of an e-cigarette. The GP/practice nurse will be encouraged to do the following during advice giving: describe the offer positively, establish the evidence-base for e-cigarettes, emphasise switching from smoking and alleviate any health concerns about the risks of vaping. Each practitioner will be given a prompt sheet as a reminder.

The GP/practice nurse will ask the participant whether s/he would be willing to try an e-cigarette, upon which the participant will be given an e-cigarette starter pack and an accompanying practical support booklet. The starter pack will contain three different flavoured bottles of e-liquid in two nicotine strengths (2 × 18 mg/ml and 1 × 12 mg/ml). Participants will need to purchase their own e-liquid thereafter. They will also receive an introductory call from an experienced vaper who will help with any technical queries they might have when first using the device. They will have the option to opt out of receiving this call.

At the end of the consultation, practitioners will record whether brief advice about e-cigarettes was given and whether the e-cigarette starter pack was accepted.

##### Practical support booklet, telephone support service and online tutorials

Intervention group participants will have access to three resources to promote patient self-efficacy, skills and positive outcome expectations about e-cigarettes, should they accept the offer of a starter pack. The first resource is a practical support booklet and will include the following: information on what is an e-cigarette, instructions on how to set up the device, how to refill and charge it, correct ways to vape, a list of local shops that sell e-liquids and common issues with use. The booklet will contain motivational support to reinforce practitioners’ advice about e-cigarettes, including the benefits of cutting down on cigarettes through e-cigarette use and addressing perceived risks and concerns about first use.

The second resource available to participants is telephone support. The practical support booklet will include the contact details of a dedicated team of experienced vapers who will operate the telephone support service. Although the vape team will have specialist knowledge on e-cigarette use, they will be trained in the study procedures and protocol for delivering technical support on e-cigarettes by staff at the University of Oxford. Each member of the vape team will be assigned 10–15 participants to assist. The study team will identify which participants accepted an e-cigarette starter pack, after the annual review appointment. If the participant accepted, his/her contact details will be passed on to a member of the vape team. The vape team will contact each participant to introduce themselves and offer any assistance on the set up and use of the e-cigarette device within one week of randomisation. Subsequently, participants will be able to contact the vape team through a telephone support service; they can leave a message for the vape team, who will return the call if they are unable to take the call immediately. All calls will be audio-recorded via a call-recording app on the study mobile phone with the consent of participants to monitor the advice provided by the vape team.

The practical support booklet will also include details of a study-dedicated website featuring a series of online video tutorials. The videos will include instructions on how to use the e-cigarette, demonstrations with a non-vaper and a testimonial from a vaper that switched from smoking to e-cigarettes.

### Practitioner training

The training will build on practitioner motivation, skills, self-efficacy and positive outcome expectancies for encouraging adherence to the protocol and delivery of the intervention [54]. LifeGuide, an open source software platform, will be used to develop and deliver the training online [57]. LifeGuide will allow us to control all aspects of the design, making changes where necessary during development and allow us to evaluate the usability and acceptability of the training.

We will draw on quantitative research to collate the evidence-base on the use of e-cigarettes for harm reduction, its use as a cessation aid and its safety profile to form part of the educational component of the training. This information—derived from sources such as the systematic review on e-cigarettes for reduction and cessation [39] and the Public Health England (PHE) evidence update [50]—will be used to develop a set of ‘key facts’ on the benefits of cutting down in smokers with chronic diseases. We will address practitioners’ main concerns about e-cigarettes, which have been reported elsewhere [48]. We will also draw on existing training resources offered to stop smoking practitioners from the National Centre for Smoking Cessation Training website. We will convey what is known and unknown about e-cigarettes and their place in treating smokers with no desire to quit. This will be followed by training to use a few key evidence-based behavioural methods from the behaviour change technique (BCT) taxonomy [58] (e.g. problem solving, instruction on how to perform a behaviour, verbal persuasion to boost self-efficacy) that practitioners will use to motivate hardcore smokers in the intervention group to try e-cigarettes if they decline help to stop smoking. This section will include video role-play scenarios between GPs and actors to show the skills in action in different settings. We will explain how important it is to trial integrity not to contaminate the study by offering e-cigarettes and advice to the control group. The training will outline the difference between what constitutes the standard care for the control group and the procedure to follow in the intervention group.

Practitioners will be given login details to access the training before the start of the intervention period. The study team will check that the training has been carried out and completed prior to delivery. If there is any uncertainty about the content and procedure, practitioners will be able to contact the study team for clarification.

### Embedded qualitative study

Participants in the intervention group will be asked whether they would be willing to participate in a telephone interview after the 2-month follow-up visit. We will purposively select up to 40 participants who took up the offer and tried an e-cigarette, those who reduced and/or quit and those who refused to use them. A semi-structured interview guide will be used to assess patients’ knowledge of e-cigarettes, their experiences of use and the degree to which the information provided by their practitioner made a difference to them and their smoking behaviour. Additionally, we will explore patients’ views on acceptability and appropriateness of being given advice and an offer of an e-cigarette by their practitioner.

We will conduct telephone interviews with up to 20 practitioners after they have seen their last enrolled patient to assess what worked well, any challenges faced when delivering the intervention and the appropriateness of the training received. We will also approach members of the vape team after they have completed their involvement in the study on what worked well and any challenges faced in providing technical support to participants in the trial. All interviews with patients, practitioners and the vape team will be audio-recorded and transcribed.

### Trial outcome measures

#### Primary outcomes

The first primary outcome is 7-day point-prevalence abstinence from smoked tobacco at 2 months. Abstinence is defined as complete self-reported abstinence from smoking—not even a puff—in the past 7 days, accompanied by a salivary anabasine concentration of < 1 ng/ml. If there are technical issues with the analysis of saliva samples (e.g. if there is not enough saliva present in the sample for anabasine analysis), we will use exhaled CO as verification of abstinence (CO < 10 ppm).

The second primary outcome is reduction in cigarette consumption at 2 months. Reduction is defined as a 50% reduction in self-reported cigarettes per day on each of the last 7 days at 2 months compared with baseline consumption, accompanied by evidence of reduced smoke intake indicated by salivary anabasine concentrations lower than baseline.

#### Secondary outcomes

Secondary outcome measures for abstinence include 7-day point prevalence abstinence measured at 8 months, biochemically confirmed by an exhaled CO of < 10 ppm. We will also measure 6-month prolonged abstinence using the Russell standard criteria, defined as smoking fewer than five cigarettes between 2- and 8-month follow-ups, confirmed by an anabasine concentration of < 1 ng/ml at 2 months and an exhaled CO concentration of < 10 ppm at 8 months (or anabasine concentration of < 1 ng/ml at 8 months if CO measurement unavailable). There is no funding for analysing all saliva samples at 8 months; therefore, we will verify abstinence using exhaled CO in the first instance.

As an additional measure of smoking reduction, we will assess the mean change in salivary anabasine concentrations from baseline to 2 months.

#### Other measures and exploratory outcomes

Baseline assessments are detailed in Table 1. Ethnic group will be classified using UK census 2015 categories. The researcher will collect a saliva sample and measure exhaled CO using a CO monitor as a measure of smoke intake. Severity of nicotine dependence will be measured using the six-item Fagerstrom Test for Cigarette Dependence (FTCD). The FTCD will be re-administered at 2- and 8-month follow-ups. We will assess motivation to stop smoking with the question “How likely are you to quit smoking within the next six months?” Responses will be measured on a five-point Likert scale from “Very unlikely” to “Very likely”.

Recruitment and follow-up outcomes will be measured by recording the number of people in the population of interest who respond to letters of invitation from the practices, the number of people who consent to enrolment into the study at baseline and the number of people who complete follow-up at 2 and 8 months after receiving the brief advice intervention.

GPs or nurses will record on the randomisation card whether an e-cigarette starter pack was accepted or declined by participants; this will be returned to the researcher after the consultation. Use of e-cigarettes will be assessed in a questionnaire administered at 2- and 8-month follow-ups. Questionnaire items include the total number of times the e-cigarette was used per day (i.e. ‘sessions’ not puffs), number of days using the e-cigarette, strength of nicotine e-liquid used and frequency of e-liquid purchased. Saliva samples collected at baseline and two-month follow-up will be analysed for cotinine, a measure of nicotine intake, arising from both e-cigarette and tobacco use. We are measuring cotinine to examine whether participants in the intervention group cut down or replace their nicotine intake with e-cigarette use.

We will also measure the number of people who tried to stop smoking, uptake and use of the NHS SSS and use of pharmacotherapy. Adverse event data will be recorded in the case report form (CRF) at 2- and 8-month follow-ups and at other points if researchers are made aware.

We will measure practitioner fidelity to the intervention using a study-specific behaviour checklist, which will be checked against audio recordings of the consultations after each session of annual review appointments. The checklist will assess whether the GP or practice nurse asked the participant about their smoking status, whether they gave brief smoking management and cessation advice, whether they offered a referral and carried out the consultation according to the correct group allocation (to assess contamination). We will draw on conversation analytic (CA) methods to inform this objective. Contamination will also be assessed in the post-consultation questionnaire, where all participants will be asked to report on whether e-cigarettes and other smoking cessation aids were mentioned during the consultation.

To measure acceptability of the intervention, we will examine participants’ views about the care they have received on their smoking in both arms by questionnaire immediately after the consultation and through semi-structured interviews with the intervention group only. Participants will be asked to rate how helpful and appropriate they found practitioners’ advice and support on smoking. They will also be asked about the quality of advice given via recall of key points of the consultation, which will be triangulated with audio recordings of the consultations. At 2 months, participants in the intervention arm will be asked whether they used the study-dedicated telephone support service, the practical support booklet and online video tutorials; they will rate each resource on a five-point Likert scale from “Very unhelpful” to “Very helpful”. We will assess attitudes towards e-cigarettes, cutting down and stopping smoking in a questionnaire administered at baseline and 2 months, as a measure of self-efficacy and outcome expectations.

Before intervention training begins and after all therapeutic visits have been completed at a practice, practitioners will be given a questionnaire about their attitudes regarding e-cigarettes as a tool for smoking cessation, as a measure of self-efficacy and outcome expectations. Practitioners and the vape team will be interviewed about their experiences of the programme after completing the trial, as described in the embedded qualitative study.

### Safety reporting

#### Adverse events

This is not a trial of an investigational medicinal product. E-cigarettes are a consumer product and the devices used in the trial are readily available to purchase in stores and online. For the purposes of the trial, we will record adverse events in the case report form by asking the participant to complete a symptoms checklist at the 2-month follow-up appointment. The checklist will contain symptoms commonly reported in previous studies on e-cigarettes [39], including throat/mouth irritation, cough, dry mouth, shortness of breath, headache, nausea, dizziness, stomach pain and palpitations. Participants will rate whether they have been troubled by any of these symptoms in the past 24 h, on a five-point Likert scale from “Not at all” to “Extremely”.

In this population, we expect that serious adverse events such as hospitalisation and death will occur during the 8 months of observation. The Cochrane review provides evidence that e-cigarettes do not cause serious adverse events but the data are sparse and imprecise [39]. As such, there are no expected serious related adverse events in this study. However, to provide further evidence on this, we will collect data on the occurrences of serious adverse events at each follow-up. We will assess each SAE in a blinded manner for relatedness to the intervention by the chief investigator (CI) for monitoring patient safety. For the purpose of trial reporting, however, we will convene an independent committee to make this judgement. Reports of related and unexpected SAEs (RUSAEs) will be reported to the REC within 15 working days of the chief investigator becoming aware of the event.

### Trial closure

The end of study is the date of the last debrief of the last participant following the 8-month follow-up.

### Statistics and analysis

#### Sample size

In order to correct the type 1 error rate for the fact that there are two primary outcomes we will be using the Holm-Bonferroni method of adjustment [59]. The smaller *P* value will be compared to an alpha of 0.025 (this will probably be for reduction in smoking) and if this is significant, the larger *P* value (which will probably be for cessation) will be compared to an alpha of 0.05. A total sample size of 320 will allow us to detect a risk ratio of 2.8 for the proportion of patients reducing their smoking and a risk ratio of 3.4 for the proportion of patients who stop smoking [38]. This assumes power of 90% and control group rates of 5% and 8% for stopping and reducing, respectively [38]. No allowance will be made for loss to follow up as all such cases will be assumed to have continued their baseline level of smoking.

We expect to see adherence to the behavioural intervention by practitioners of 70%, use of the e-cigarette at follow-up of 40%, and follow-up 70% of enrolled participants. We will be able to estimate these proportions with 95% confidence intervals being no greater than ±8% with a sample of 160 people in the intervention group and ±6% for the whole sample of 320 participants.

### Analysis plan

#### Primary and secondary analyses

The proportion of participants who reduce smoking in each group will be presented. A log-binomial regression model will be applied to the data and the treatment effect will be reported as a relative risk and 95% confidence interval, adjusted for GP practice. A similar procedure will be used to analyse the proportion of participants who abstain from smoking. Analyses will be carried out on an intention-to-treat basis, according to the Russell Standard [60], where participants lost-to-follow up are assumed to be smokers or not to have reduced and we will impute missing anabasine concentrations with the baseline value.

The mean (SD) change in salivary anabasine from baseline to 2 months will be reported for each group and the difference and 95% CI will be computed using linear regression with adjustment for GP practice. Assumptions of linear regression will be assessed and if violated a Mann-Whitney test will be adopted and the median (IQR) will be used to summarise the data and difference in medians (95% CI) will be reported.

#### Other analyses

For examining uptake and use of e-cigarettes, we will calculate the proportion of participants who take up the offer of an e-cigarette and the proportion who continue to use them at 2- and 8-month follow-ups. Proportions will be reported using descriptive statistics. Continued use of e-cigarettes will mean either use until complete smoking cessation was achieved or continued use on > 50% days between baseline and follow-up. For changes in nicotine intake, we will calculate the mean change in salivary cotinine concentrations from baseline to 2-month follow-up for each group and the difference and 95% CI will be computed using linear regression with adjustment for GP practice. Assumptions of linear regression will be assessed and if violated a Mann-Whitney test will be adopted and the median (IQR) will be used to summarise the data and difference in medians (95% CI) will be reported.

We will compare the proportion of people who consent to enrolment into the study and complete 2- and 8-month follow-ups. We will examine these outcomes separately at each recruitment centre to assess the generalisability of our recruitment procedures. Proportions will be reported using descriptive statistics and the difference between trial arms and 95% CI will be calculated.

For participants’ views of the programme (assessed by Likert scale questions), we will calculate the total score and compare these by trial arm using linear regression with adjustment for GP practice. Participants’ recall of key points of the consultation (reported as a yes/no variable) will be presented as frequencies and compared between the two arms using a chi-squared test.

Practitioner fidelity to the intervention will be assessed using a rating scale for key elements in the behavioural interventions and summarised as a score. These are descriptive process data and relate only to the intervention group.

The patient, practitioner and vape team interview analysis will follow the Framework approach [61], categorising data into cases and coded themes. We will follow an inductive approach, drawing on participants’ perspectives and experiences of the intervention to develop a coding matrix. Interviews will be analysed as the data are collected so that insights from initial data analysis can be explored in later interviews and used to refine themes and categories. We will carry out this process until data saturation is reached, where no new themes emerge. Data will be analysed using NVivo software.

We will analyse intervention delivery during the consultations using conversation analysis (CA), a well-established qualitative methodology used to analyse the sequential organisation of verbal and non-verbal aspects of talk-in-interaction [62]. CA has been extensively applied in routine healthcare, particularly in general practice settings [63], and is now beginning to be applied to the analysis of talk-based healthcare interventions in RCTs [64, 65]. The aim is to uncover differences in the communication practices used by clinicians that seem more or less effective at persuading participants to take and try using e-cigarettes. We will transcribe and analyse a sample of 50 audio-recordings using this technique, firstly by identifying the consultations that resulted in patients trying an e-cigarette and comparing communication practices and patient uptake across practitioners. Most communication training uses instruction or role-play but this often fails to give insight into the complexity of real-life encounters. Real examples of ‘trainables’ from the CA analysis, highlighting ‘what works’, i.e. the kind of interactional issues that can occur during intervention delivery and the techniques that can best resolve them, will be collected to inform future work. By doing so we will be able to demonstrate both ‘plausible framings and efficacious implementations’ of our intervention, and ways in which communication behaviour change can be incorporated into an existing clinical skillset [66].

### Data handling, record keeping and retention

The trial is being run as part of the portfolio of trials in the Primary Care Clinical Trials Unit (PC-CTU) at the University of Oxford. The data management will be run in accordance with the Trials Unit standard operating procedures (SOPs), which are fully compliant with Good Clinical Practice (GCP). A study-specific Data Management Plan (DMP) will be developed for the trial outlining in detail the procedures that will be put in place to ensure that high quality data are produced for statistical analysis.

A unique trial-specific number and/or code in any database will identify the participants. All data will be directly entered into electronic case report forms (eCRFs). A full pre-entry review and electronic data validation for all data entered into the clinical database will be provided by study-specific programmed checks. All paper data will be locked in secure cabinets and only the researchers will have access to the files. On all trial-specific documents, other than the signed consent, the participant will be referred to by the trial participant number/code, not by name. A separate database will be used to securely store all identifiable patient information required to contact patients and permit follow up. Access to this information will be strictly on a need to know basis.

On completion of the trial and data cleaning, the study documentation will be transferred to a secure, GCP-compliant archiving facility, where they will be held for 5 years. Participants’ identifiable information will be destroyed at the end of the trial. Prior to database lock, the Data Manager and the Trial Statistician will undertake a dataset review. Following this, quality control will be performed on queries relating to critical data items by the Data Manager.

#### Sample handling

Researchers will obtain saliva samples from participants at baseline and 2-month follow-up visits to measure salivary cotinine and anabasine concentrations. Cotinine, a major metabolite of nicotine, has a half-life of approximately 20 h in smokers [67] and is stable at room temperature for up to 12 days [68]. Anabasine, a specific biomarker of tobacco smoke, has a half-life of 16 h [69] and is also stable at room temperature for up to 14 days.

Saliva will be collected in a plastic vial containing a sterile dental roll (Salivette, Sarstedt). The container will be labelled with the patient ID, type of sample, collection date and time, then transported back to the university. Samples will be sent to the laboratory as soon as they are collected, or they may be stored for up to 7 days at room temperature until they are shipped in batches to a laboratory. The laboratory will store and freeze the samples at − 20 °C until there is a sufficient batch for processing. Salivary cotinine and anabasine concentrations will be determined by liquid chromatography-tandem mass spectrometry (LC-MS/MS) assay. If insufficient saliva is available in the sample for both cotinine and anabasine analysis, anabasine will take priority in the assay. All samples will be disposed of by laboratory staff after final analysis.

### Quality assurance procedures

The trial will be conducted in accordance with the current approved protocol, GCP, relevant regulations and standard operating procedures.

Regular monitoring will be performed according to GCP. Data will be evaluated for compliance with the protocol and accuracy in relation to source documents. Following written standard operating procedures, the monitors will verify that the clinical trial is conducted and data are generated, documented and reported in compliance with the protocol, GCP and the applicable regulatory requirements.

An independent Trial Steering Committee (TSC) will provide oversight of all matters relating to participant safety and data quality. The TSC will include at least one independent clinician, an independent statistician and a participant representative. The TSC will be asked to review the trial protocol and will provide expert advice to the Trial Management Group on the trial progress.

A data monitoring committee is not required for this study as it is of low risk and the trial protocol will not be modified based on interim data.

### Patient and public involvement

The study is supported by a patient advisory group who are involved in several aspects of the trial, including the design, management and conduct of the trial through membership of the trial steering committee. Members include active smokers from our target population, smokers trying to quit and ex-smokers, some of whom have used or are currently using e-cigarettes. Additionally, one member of the group is an experienced vaper and will lead the telephone support service that will provide technical support to patients in the trial on e-cigarette use. The group will help develop and review the practical support booklet, online content and lay summaries of the study findings.

### Expenses and benefits

As there is no therapeutic benefit to participants for attending the baseline and follow-up appointments in person at 2 and 8 months, we will pay those participants who attend a reimbursement (£30 voucher) to cover the cost and inconvenience of attending. Patients who are invited to take part in post-follow-up interviews will be reimbursed £20. For participating in interviews, GPs will be reimbursed £80 per hour, practice nurses will be reimbursed £21.96 per hour and members of the vape team will receive £20 reimbursement.

## Discussion

The heart of this intervention is a brief intervention from the GP or practice nurse to patients to advise them that using an e-cigarette is safer than continuing to smoke and may help them cut down their smoking. That their GP or practice nurse believes this to be true would be effectively communicated by a consent procedure that fully outlined the nature of both interventions; however, this would break the trial randomisation. Instead, to reduce contamination, we will inform potential participants that this is a trial about the way practitioners have conversations with people about their smoking. This approach may also reassure people who have no plans to stop smoking that joining the study will not involve them being subject to hectoring to do something they do not want to do. We will ensure that all patients are fully debriefed and are informed about the purpose of the study at the end of the trial with a debriefing information sheet.

If practitioners can be trained to offer advice about e-cigarettes and patients take up this offer, patients at high risk of increased morbidity and mortality from smoking could be helped to reduce their smoking and increase their chances of quitting. For almost all these specified smoking-related conditions, stopping smoking would be the single most effective way to improve health for patients and is likely to reduce health service costs.

## Trial status

Recruitment began in June 2018. At the time of manuscript submission, the trial was still in the recruitment phase, with 243 participants recruited as of 1st April 2019.

At the time of manuscript resubmission, recruitment had finished in May 2019 and the trial was in follow-up. This manuscript is based on version 3.0 (21 May 2019) of the study protocol.

## Supplementary information

**Additional file 1.** Objectives, outcomes and time-points.

**Additional file 2.** SPIRIT checklist.

**Additional file 3.** Participant decision enrolment tree.

**Additional file 4.** Patient information sheet.

**Additional file 5.** Patient consent form.

## Data Availability

The study materials supporting this paper are included as additional files.

## Abbreviations

CI: Chief investigator
CO: Carbon monoxide
COPD: Chronic obstructive pulmonary disease
CRF: Case report form
DMP: Data management plan
eCRF: Electronic case report form
FTCD: Fagerstrom test for cigarette dependence
GCP: Good clinical practice
GDPR: General Data Protection Regulation
GP: General practitioner
HRA: Health Research Authority
LC-MS/MS: Liquid chromatography-tandem mass spectrometry
NCSCT: National centre for smoking cessation training
Ng/ml: Nanograms per millilitre
NHS: National health service
NICE: National Institute for Health and Care Excellence
NIHR: National Institute for Health Research
NRT: Nicotine replacement therapy
PC-CTU: Primary care clinical trials unit
PHE: Public Health England
PIS: Patient information sheet
ppm: Parts per million
QOF: Quality and outcomes framework
RCT: Randomised controlled trial
REC: Research ethics committee
SAE: Serious adverse event
SMI: Serious mental illnesses
SSS: Stop smoking services
SOP: Standard operating procedure
TSC: Trial Steering Committee
