# Supplementary material for: Examining the effectiveness of general practitioner and nurse promotion of electronic cigarettes versus standard care for smoking reduction and abstinence in hardcore smokers with smoking-related chronic disease: protocol for a randomised controlled trial
Source: Trials. 2019 Nov 28;20:659. doi: 10.1186/s13063-019-3850-1 (PMC6883522; doi:10.1186/s13063-019-3850-1)
Supplement: Supplementary file 1 — Additional file 1. Objectives, outcomes and time-points. [file 13063_2019_3850_MOESM1_ESM.docx]

| **Objectives** | **Outcome Measures** | **Timepoint(s) of evaluation of this outcome measure (if applicable)** |
| --- | --- | --- |
| **Primary Objective** To examine the effectiveness of a brief GP/nurse behavioural intervention to encourage switching to e-cigarettes, provision of e-cigarettes, and ongoing technical support from experienced e-cigarette users in producing short-term reductions in cigarette intake and smoking abstinence. | - Smoking reduction measured through change in cigarettes per day confirmed by reduction in salivary anabasine concentrations. Reduction is defined as a 50% reduction in cigarettes per day on each of the last seven days at two month follow-up compared with baseline consumption, accompanied by evidence of reduced smoke intake indicated by salivary anabasine concentrations lower than baseline. - 7-day point-prevalence abstinence at two month follow-up accompanied by an anabasine concentration that verifies this (<1ng/ml). | - Saliva samples collected at baseline and at two month follow-up - Saliva samples collected at baseline and at two month follow-up |
| **Secondary Objectives**  To examine recruitment and follow-up of patients  To examine smokers’ uptake and use of offered e-cigarettes.  To assess contamination of randomisation  To examine nicotine intake  To assess the adherence of primary care teams in delivering brief interventions  To examine practitioners’ attitudes and reactions towards offering e-cigarettes and experiences of delivering the intervention  To examine patients’ attitudes and reactions to the programme  To examine the vape team’s reactions towards supporting patients in their use of e-cigarettes  To examine the effectiveness of a GP/nurse-led brief intervention for smoking on long-term reductions in cigarette intake and smoking abstinence | - Proportion of people who respond to letter of invitation, the proportion who meet eligibility and consent to enrolment into the study and complete follow-up - Proportion of people who take up the offer of an e-cigarette and the proportion of people who continue to use them. - GPs give the intervention to people in the control group - Nicotine intake as measured by the mean change in salivary cotinine concentrations - Practitioner fidelity measured using a study-specific checklist of behavioural techniques via analysis of recorded consultations - Practitioners’ views about the programme explored through semi-structured interview and attitudes towards e-cigarettes assessed via pre-training and post-intervention questionnaire - Participants’ views about the programme assessed via post-consultation questionnaire and semi-structured interview; attitudes assessed via questionnaire - Vape team’s views about the programme assessed via semi-structured interview - 7-day point-prevalence abstinence at eight months and prolonged abstinence from two months, verified by exhaled CO measurement - Reduction in cigarettes per day at eight months on each of the last seven days compared with baseline consumption | - Recruitment and follow-up measured at baseline and via completion of two and eight month follow-ups - Post-consultation questionnaire, questionnaire at two and eight month follow-ups - Post-consultation questionnaire and intervention recordings. - Saliva samples collected at baseline and at two month follow-up - Recordings collected after each annual review session during 12 month intervention period - Interviews conducted after participating in the trial and questionnaire given pre-training and post-intervention - Post-consultation questionnaire and interviews at two month follow-up; attitudes questionnaire at baseline and two month follow-up - Interviews conducted after participating in the trial - Exhaled CO or saliva sample measurement at eight months - Self-reported cigarettes per day at eight months |
