# Supplementary material for: Examining the effectiveness of general practitioner and nurse promotion of electronic cigarettes versus standard care for smoking reduction and abstinence in hardcore smokers with smoking-related chronic disease: protocol for a randomised controlled trial
Source: Trials. 2019 Nov 28;20:659. doi: 10.1186/s13063-019-3850-1 (PMC6883522; doi:10.1186/s13063-019-3850-1)
Supplement: Supplementary file 3 — Additional file 3. Participant decision enrolment tree. [file 13063_2019_3850_MOESM3_ESM.docx]

**YES – patient annual review booked with GP/nurse**

Patient ineligible for study

**NO**

Patient ineligible for study

Patient randomised to intervention group

Patient randomised to control group

**YES – GP/nurse enrols and randomises patient**

**YES – usual care smoking cessation treatment provided by GP/nurse**

**YES - patient invited to baseline visit**

**NO**

**NO**

Patient ineligible for study

Patient ineligible for study

Does the patient meet initial eligibility during telephone screening?

Does the patient meet eligibility criteria at baseline visit?

Is the patient still smoking at the therapeutic visit?

Did the patient decline usual care smoking cessation treatment?

**NO**
