# Supplementary material for: Examining the effectiveness of general practitioner and nurse promotion of electronic cigarettes versus standard care for smoking reduction and abstinence in hardcore smokers with smoking-related chronic disease: protocol for a randomised controlled trial
Source: Trials. 2019 Nov 28;20:659. doi: 10.1186/s13063-019-3850-1 (PMC6883522; doi:10.1186/s13063-019-3850-1)
Supplement: Supplementary file 4 — Additional file 4. Patient information sheet. [file 13063_2019_3850_MOESM4_ESM.docx]

**
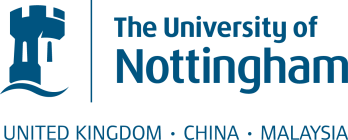
**
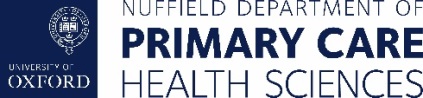


**Find out more about our study!**

**
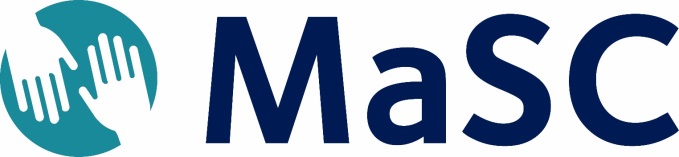
**

**Participant Information Sheet**

What is this study about?

- We need **your help** in learning about **the best way** your GP and Nurse can talk to patients who don’t want to stop smoking
- We want to **understand** whether people find it helpful if their GP or Nurse talks to them about smoking in one of two ways.
- Before you decide if you would like to take part, we would like you to understand why the research is being done and what it would involve for you.


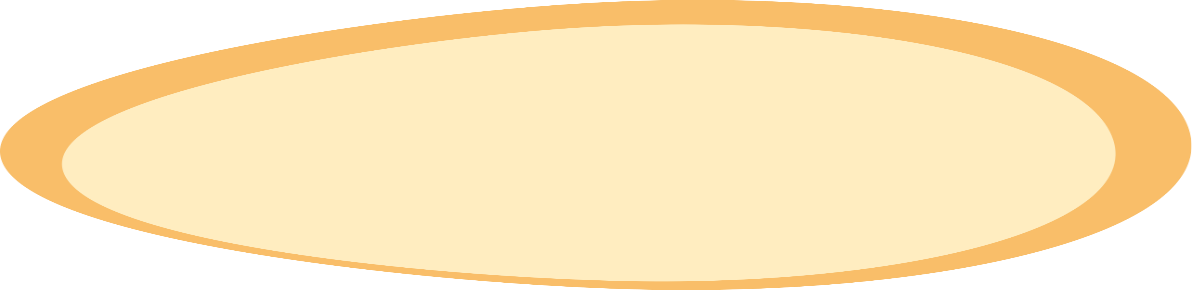


**OUR CONTACT DETAILS:**

**[insert details according to centre]**

**University of Oxford University of Nottingham**

**Address:** xxxxxx **Address:** xxxxxx

**Tel:** xxxxxx **E-mail:** xxxxxx **Tel:** xxxxxx **E-mail:** xxxxxx

Receive at least a **£30** gift voucher as a

thank you for your time!

Why have I been chosen?

Because your medical notes say that you smoke and that you have a medical condition. If this isn’t true, please feel free to ignore this letter.

What will I have to do?

**Visit 1 – with our researcher**

- Meet our researcher at your GP surgery
- Answer some questions about yourself and cross-refer to your medical records
- Provide a saliva sample so that we can look at how much you currently smoke
- Do a quick breath test to check for carbon monoxide in your lungs

The first visit will last between 30-45 minutes and **you will receive a £30 voucher for your time**.

**Visit 2 – with your GP or Nurse**

Each year your GP or nurse talks to you and examines you to check on your medical condition. As part of this check-up, the GP or nurse gives you advice about smoking. This visit will take place anyway whether you take part in our study or not.

If you do take part, we will ask the GP or nurse to give you advice that may be different from usual. Whether the GP or nurse gives you usual advice or the different advice will depend upon the content of an envelope, which will tell the GP or nurse which kind of advice to give. Neither you nor your GP or nurse will know what is in the envelope because this has been decided randomly (like tossing a coin). The GP or nurse will ask for your permission to record the consultation because we would like to know how your GP or nurse is delivering the advice, but you can opt out of this.

After the appointment, the GP or nurse will ask you to see a researcher in another room to ask you a couple of questions about what you thought of the discussion you had with your GP or nurse. This will take 2 minutes or less.

**Visit 3 – with our researcher**

We will ask you to meet our researcher at your GP surgery 2 months after you have seen your GP or nurse. Here you will:

- Answer some questions about yourself and cross-refer to your medical records
- Provide a saliva sample so that we can look at how much you smoke
- Do a quick breath test to check for carbon monoxide in your lungs

The visit will last around 30 minutes and **you will receive a £30 voucher for your time.**

**Visit 4 – telephone call and possibly a visit to our researcher**

We will call you 6 months later to ask you some questions about yourself. This will take less than 5 minutes. We may ask to see you again at your GP surgery for a breath test. This visit will take 5 minutes and **you will receive a £30 voucher for your time.**

**Optional extra visit or telephone call**

We will ask to spend about 30 minutes talking to some people who take part in the study, which will be recorded. This is to find out about what they thought about the discussion they had with their GP or nurse. If we do ask to chat with you, **you will receive a £20 voucher for your time.**

Are there benefits or risks of taking part?

There are no known risks to taking part in the study. What we learn from this study could help us train GPs to give better advice to people who smoke in the future. As part of your routine check-up, your GP or nurse will offer you medication or other devices to help with your smoking. You could decide not to use this, but if you do use it, there are some known side-effects and your GP or nurse will talk to you about the benefits and risks so you can decide at the time.

What if I don’t want to be involved anymore?

You can leave the study at any point and the decision to do so will not affect the treatment you receive from your GP or nurse. We would still like to use the data you have already provided, as this will be invaluable to our research. If you have any objection to this please let us know.

What if there are any issues?

For queries about this trial, please contact the study team on xxxxxx or email xxxxxxx.

If you wish to complain about any aspect of the way in which you have been treated during the trial, you should contact the chief investigator, Dr Rachna Begh, or the trial co-ordinator, or the University of Oxford Clinical Trials and Research Governance (CTRG) office on 01865 572224 or email [ctrg@admin.ox.ac.uk](mailto:ctrg@admin.ox.ac.uk).

The University of Oxford, as Sponsor, has appropriate insurance in place in the unlikely event that you suffer any harm as a direct consequence of your participation in this study. NHS indemnity operates in respect of the clinical treatment with which you are provided.

What about confidentiality?

All data will be kept securely according to the Data Protection Act 1998. All trial information collected will be made anonymous at the earliest practical opportunity. The information you provide at the first consultation and subsequent appointments, will be coded with a trial identification number so you cannot be identified from it by anyone other than the research team. If we use any quotations and audio clips in publications or training materials, these will be made anonymous.

What will happen to the results?

The results of this research study will be published in a scientific medical journal and on the University website and may be used in future training materials. Data may also be used by students as part of an educational project. Your individual results will not be identifiable nor would you be identified in any report or publication.

Who funded and approved the study?

This trial is being funded by the National Institute for Health Research. It has been reviewed and given favourable opinion by South Central Oxford xxx Research Ethics Committee (ref xxxxxx).

**Thank you for considering**

**taking part in this trial.**
