# Supplementary material for: Examining the effectiveness of general practitioner and nurse promotion of electronic cigarettes versus standard care for smoking reduction and abstinence in hardcore smokers with smoking-related chronic disease: protocol for a randomised controlled trial
Source: Trials. 2019 Nov 28;20:659. doi: 10.1186/s13063-019-3850-1 (PMC6883522; doi:10.1186/s13063-019-3850-1)
Supplement: Supplementary file 5 — Additional file 5. Patient consent form. [file 13063_2019_3850_MOESM5_ESM.docx]

**Chief Investigator: Dr Rachna Begh**

Department of Primary Care Health Sciences

The University of Oxford

[Rachna.begh@phc.ox.ac.uk](mailto:Rachna.begh@phc.ox.ac.uk)

Tel: +44 (0) 1865 617 191

| **PATIENT INFORMED CONSENT FORM** | **Please initial each box** | | |
| --- | --- | --- | --- |
| **Study Title**: MaSC ( **Ma**nagement of **S**moking in Primary **C**are) |  | | |
| 1. I confirm I have read and understood the information sheet dated (Version ………………) for the MaSC study and have had the opportunity to ask questions and had these answered satisfactorily. |  |  |  |
|  |  |  |  |
| 2. I understand that my participation is voluntary and that I am free to withdraw at any time, without giving any reason, and without my medical care or legal rights being affected |  |  |  |
|  |  |  |  |
|  |  |  |  |
| 3. I understand that relevant sections of my medical notes and data collected during the study may be looked at by individuals from the University of Oxford and University of Nottingham, from regulatory authorities or from the NHS Trust, where it is relevant to my taking part in this research. |  |  |  |
|  |  |  |  |
| 4. I understand that my anonymised data may be shared with other researchers for research purposes only. I permit these individuals access to my research records. |  |  |  |
|  |  |  |  |
| 5. I agree to donate saliva samples. I consider these samples a gift to the University of Oxford and understand that I will not gain any personal or financial benefit from them. |  |  |  |
|  |  |  |  |
|  |  |  |  |
| 6. I understand that by signing this consent form the information given to researchers will be transferred to Oxford University and stored securely. |  |  |  |
|  |  |  |  |
| 7. I agree to audio recording and the use of anonymised quotes in research reports and publications. I understand that my audio recording will be sent to an external transcription company for transcribing. *(Optional)* |  |  |  |
|  |  |  |  |
| 8. I agree to take part in a more in depth discussion about my experiences of being part of the MaSC study and that this will be audio-recorded and sent to an external transcription company for transcribing and analysed by researchers. I understand that my anonymised quotations may be used in publications and training materials. *(Optional)* |  |  |  |

| 9. I agree to take part in the above study. | | | | | | | | |  |  | | |  |  |
| --- | --- | --- | --- | --- | --- | --- | --- | --- | --- | --- | --- | --- | --- | --- |
|  | | | | | | |  | |  | |  |  | | |
|  | | | | | | | | | | | | | | |
|  |  |  | |  |  |  |  |  |  |  |  |  |  |  |
|  | | |  |  |  |  |  |  |  |  |  |  |  |  |
| PRINT Participant name | | | | |  | Participant signature | |  | Date | | | | |  |
|  | | | | |  |  | |  |  | | | | |  |
| PRINT Researcher name | | | | |  | Researcher signature | |  | Date | | | | |  |

**1 copy for participant; 1 copy for researcher site file; 1 copy for master file*
